# Is leukopenia and lymphopenia a characteristic feature of COVID-19 in children?

**DOI:** 10.12669/pjms.37.3.3848

**Published:** 2021

**Authors:** Attia Bari, Aimen Ch, Iqbal Bano, Nazish Saqlain

**Affiliations:** 1 Dr. Attia Bari, (DCH, MCPS, FCPS, MSc-HPE) Associate Professor Pediatric Medicine Department of Pediatric Medicine, The Children’s Hospital and The Institute of Child Health, Lahore, Pakistan; 2 Dr. Aimen Ch, (FCPS), Senior Registrar, Department of Pediatric Medicine, The Children’s Hospital and The Institute of Child Health, Lahore, Pakistan; 3 Dr. Iqbal Bano, (FCPS) Associate Professor Pediatric pulmonology Department of Pediatric Pulmonology, The Children’s Hospital and The Institute of Child Health, Lahore, Pakistan; 4 Dr. Nazish Saqlain, (FCPS) Assistant Professor Hematology, Department of Pediatric Hematology, The Children’s Hospital and The Institute of Child Health, Lahore, Pakistan

**Keywords:** coronavirus disease 2019, children, acute respiratory disease, mortality, prognosis, blood picture

## Abstract

**Objectives:**

To analyze whether leucopenia and lymphopenia a characteristic feature of children with COVID-19 and to find out its association with the disease severity.

**Methods:**

This was a descriptive cross-sectional study conducted at The Children’s Hospital Lahore from March 2020 to October 2020. All confirmed cases of COVID-19 infection and post-COVID MIS-C/Kawasaki Disease diagnosed on the basis of RT-PCR and Antibody test respectively were included. Complete blood and differential counts were performed on the day of admission.

**Results:**

Out of a total of 83 patients 60 (72%) were diagnosed as COVID-19 and 23 (28%) as post-COVID MIS-C/KD. The mean age of children was 7.0±4.3 years (95%CI: 6.07 - 8.75) with a male preponderance 51 (61%). Twenty (24%) children had an underlying comorbidity and 7 (8%) were surgical cases. Our case fatality rate was 5 (6%) and all children who died had an underlying comorbid condition. In both, COVID and MIS-C/KD the mean leukocyte count was (14.0 ± 12.5 vs 13.6 ± 6.9 x10^9^/L), respectively (p=0.888). The mean lymphocyte count in children with COVID was (39.1 ± 21.4%). Patients with MIS-C/KD showed significantly higher levels of neutrophil count (76.5 ± 15.0%) as compared to children with COVID (52.0 ± 22.1%), absolute lymphocyte count was (5.02±4.81 vs 2.13±0.95 x10^9^/L) in COVID and MIS-C respectively (p=<0.001). In 60 COVID-19 patients, the mean neutrophil lymphocyte ratio (NLR) in mild-moderate and severe-critical group was 2.00 and 5.08 respectively (p=0.009).

**Conclusion:**

The blood picture of COVID-19 in children does not show leukopenia. NLR was a prognostic factor to assess the severity in COVID-19 patients. The presence of an underlying comorbid conditions is significant a risk factor for poor outcome.

## INTRODUCTION

A cluster of unexplained pneumonia cases was reported to the World Health Organization (WHO) from Wuhan, China on 31 December, 2019. The etiology for this outbreak was found to be severe acute respiratory syndrome coronavirus 2 (SARS-CoV-2) and the disease was named as Corona Virus Disease 2019 (COVID-19). The COVID-19 has been declared as a pandemic by WHO on March 11, 2020.^1^

The COVID-19 in pediatric age group is very challenging to diagnose and treat.^2^ The disease in children is generally mild and patients can be managed at home but patients having moderate or severe symptoms need hospitalization for close observation and supportive care.^3^ A large number of risk factors are known for morbidity and mortality, including age, gender, ethnicity, comorbid conditions and laboratory parameters. Old age patients and those with comorbid conditions are at increased risk of mortality from COVID-19. However, young people without any underlying disease can also present with possibly fatal complications like myocarditis and disseminated intravascular coagulopathy.^4^

In severe COVID-19, children often have abnormal laboratory parameters that reveal a systemic inflammatory response. Some of these are predictor of unfavorable clinical outcome and are evolving as reliable prognostic biomarkers. Severe disease is usually complicated by leukopenia, lymphopenia, thrombocytopenia and coagulopathy often leading to disseminated intravascular coagulopathy.^4^ In some studies, it was concluded that almost 85% of severely or critically ill patients of COVID-19 exhibit lymphopenia and there is associated high risk of ARDS and poor outcome. However, in pediatric age group data is still lacking.^5^

Researches from Pakistan on lymphocyte counts in COVID-19 in children, association between lymphocyte count and disease severity leading to ultimate outcome appear to be scarce. So, we planned our research to analyze whether leukopenia and lymphopenia is a characteristic feature of COVID-19 in children and to find out the association between these two parameters of blood counts with disease severity and outcome of COVID-19 infection in children presenting to our hospital.

## METHODS

A descriptive cross-sectional study was conducted from March 2020 to October 2020 at The Children’s Hospital and Institute of Child Health Lahore. After taking approval from hospital Institutional Review Board (manuscript no: 2020-155-CHICH) and consent from the parents, we included all children from birth to 16 years of age admitted to COVID unit and diagnosed as COVID-19 on basis positive nasopharyngeal swab for RT-PCR and post-COVID MIS-C/Kawasaki disease (KD) like illness based on positive serology for antibody and presence of clinical criteria. Suspected case who turned out to be negative for RT-PCR and serology for antibody were excluded. A predesigned proforma was used for data collection.

Based on WHO ARI criteria, the disease severity was categorized and as per WHO criteria, the diagnosis of post COVID MIS-C/ Kawasaki like illness was made. The disease was categorized as Asymptomatic: with no symptoms, Mild: respiratory tract symptoms without fast breathing, Moderate: fast breathing according to age and radiological evidence of pneumonia, Severe disease: dyspnea, hypoxia, or >50 percent lung involvement on imaging within 24 to 48 hours, Critical disease: ARDS with respiratory failure, shock, or multi-organ dysfunction.^6^,^7^ Laboratory tests including CBC with differential count and X-ray chest was done in all patients and echocardiography was done for children with MIS-C/ KD. Normal WBC count was taken as 11±5 x10^9^/L in children under 2 years of age, 2-6 years 10±5 x10^9^ /L and >6 years 9±4 x 10^9^/L. Normal Lymphocyte count in children under two years as 3.5-11 x10^9^/L, in 2-6 years 6-9 x10^9^ /L and >6 years 1-5 x 10^9^/L.^8^ Neutrophil Lymphocyte Ratio (NLR) was calculated. Outcome was declared as discharged, died or left against medical advice (LAMA).

Statistical software SPSS -24 was used for data analysis. The quantitative variables like age, WBC count and lymphocyte count were presented as mean and SD. Qualitative variables like clinical presentation, disease severity and outcome were presented as frequency and percentages. To compare continuous variables in data from different patient groups, the Independent t-test was used and One-way-ANOVA test was applied for comparing means of four different severity groups and a p-value <0.05 was considered as significant.

## RESULTS

Out of a total of 83 patients 60 (72%) were diagnosed as COVID-19 and 23 (28%) as post COVID MIS-C/ KD. The mean age of children was 7.0±4.3 years (95%CI: 6.07 - 8.75) with a male preponderance 51 (61%). Twenty (24%) children had an underlying comorbidity and seven (8%) were surgical cases (Table-I).

**Table-I:** Demographic characteristics of children admitted with COVID-19 (N= 83).

Demographic characteristics	Number (%)
Mean Age (Years)	7.0±4.3
***Age groups***	
<1 year	12 (14.5)
1-5 years	16 (19)
5-10 years	33 (40)
10-16 years	22 (36.5)
***Gender***	
Male	51 (61)
Female	32 (39)
***COVID-19/ Post-COVID***	
COVID-19	60 (72)
Post COVID/Kawasaki like illness	23 (28)
***Disease Severity***	
Asymptomatic	10 (12)
Mild-Moderate	53 (64)
Severe/ critical	20 (24)
***Comorbidity***	
Present	20 (24)
Surgical cases	07 (8)
***Outcome***	
Discharged	78 (94)
Died	05 (6)

Among the comorbid condition’s chronic kidney disease 7 (8.4%) was the commonest underlying medical condition followed by congenital heart disease 3 (3.6%), celiac disease 2 (2.4%), malignancy 2 (2.4%), immune deficiency 2 (2.4%), chronic liver disease 1 (1.2%), diabetes mellitus 1 (1.2%), cerebral palsy 2 (2.4%) and 7 (8.4%) were surgical cases. Our case fatality rate was five (6%) and four children who died had some underlying comorbid condition (p=0.019) and only one died of post COVID-MIS-C in which there was delay in diagnosis. The predominant symptom was fever followed by cough and respiratory difficulty. Details of symptoms are described in (Table-II). No direct or indirect contact was found in 34 (41%) and cluster cases (2 or more cases of fever and/or respiratory symptoms within 2 weeks) were identified at home in 8 (10%).

**Table-II:** Symptoms and Contact positivity of Children admitted with COVID-19.

Symptoms & Contact Positivity	Number (%)
Fever	62 (85)
Cough	35 (48)
Respiratory difficulty	25 (34)
Rhinorrhea	09 (12)
Poor feeding	20 (27)
Body aches	10 (14)
Vomiting	16 (22)
Loose motions	14 (19)
Abdominal pain	13 (18)
Seizures	09 (12)
***Contact with Positive COVID-19 cases***	
Direct/ indirect	49 (59)
No contact identified	34 (41)
Cluster cases of respiratory disease	8 (10)

In both groups (COVID and MIS-C/ KD) the mean leucocyte count was (14.0 ± 12.5 vs 13.6 ± 6.9 x10^9^/L) respectively (p=0.888). The mean lymphocyte count in children with COVID was 39.1±21.4%. Patients with MIS-C/KD showed significantly higher levels of neutrophil count (76.5 ± 15.0%) as compared to children with COVID (52.0 ± 22.1%), absolute lymphocyte count was (5.02±4.81 vs 2.13±0.95 x10^9^/L) in COVID and MIS-C respectively (p=<0.001) (Table-III).

**Table-III:** Complete Blood Counts in COVID-19 and MIS-C/ Kawasaki Disease.

Complete blood counts	COVID-19	MIS-C/ Kawasaki	p-value
Mean hemoglobin (g/dl)	10.6 ± 2.2	10.4 ± 1.6	0.698
Mean Leucocyte count (10^9^/L)	14.0 ± 12.5	13.6 ± 6.9	0.888
Mean Neutrophil count %	52.0 ± 22.1	76.5 ± 15.0	<0.001
Mean Lymphocyte count %	39.1 ± 21.4	18.8 ± 12.8	<0.001
Absolute Lymphocyte count (10^9^/L)	5.02±4.81	2.13±0.95	<0.001
Mean Platelet count (10^9^/L)	297.61 ± 147.3	238.5 ± 206.0	0.159

**Fig.1:**
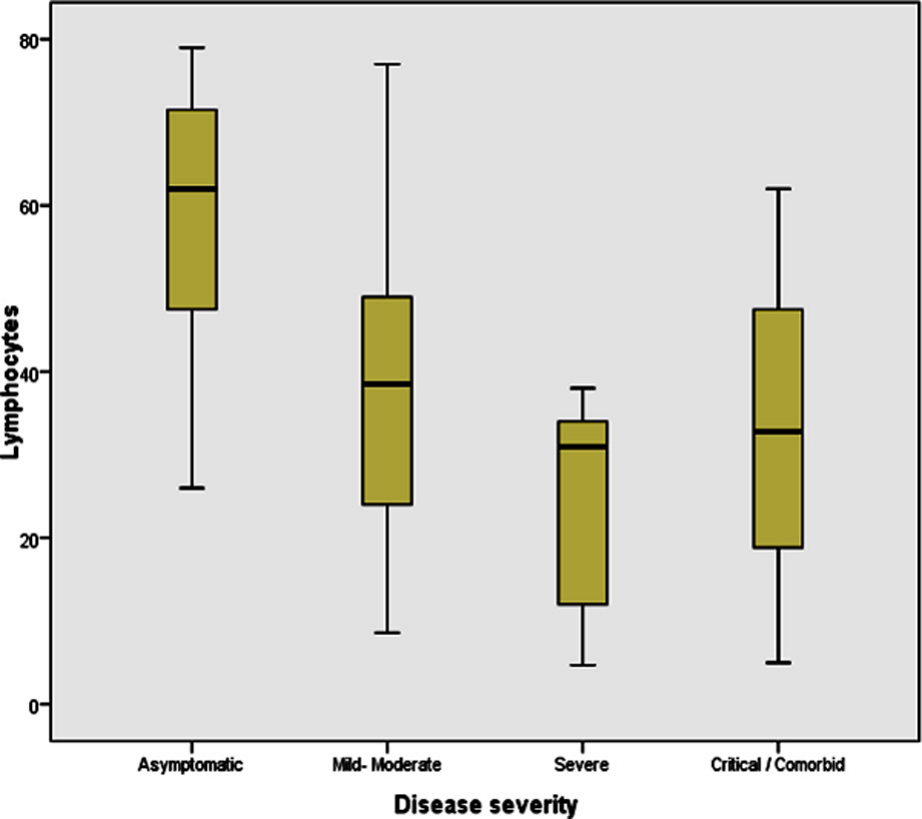
Peripheral Lymphocyte count and Disease Severity in COVID-19.

The mean NLR was 3.90 ± 4.84 and median was 1.96. In 60, COVID-19 patients the mean NLR in mild-moderate and severe-critical group was 2.00 ± 2.25 and 5.08±6.21 respectively (p=0.009). Mean differential lymphocyte count in COVID-19; asymptomatic, mild-moderate, severe and critical/ comorbid condition was 57.86±18.93, 38.84±20.14, 28.41±21.04 and 33.18±23.27 (%) respectively (p=0.043). Relationship of lymphocyte count and disease severity is shown in (Fig.1).

## DISCUSSION

Since the pandemic of coronavirus is ongoing, many studies have been published to highlight the importance of hematological abnormalities to predict the severity of disease. Many studies have shown the increased prevalence of lymphopenia in serious COVID-19 infected patients.^9^ However, when we analyzed the data of our pediatric COVID-19 positive patients and patients with MIS-C/KD we found that there was no significant leukopenia and lymphopenia at the time of admission in our patients. Literature is daily coming up on the relationship of lymphopenia and COVID-19 severity but we are still at the early stage to determine if this association of disease severity and lymphopenia really exists in children. Moreover, there is no published literature on this aspect in children with COVD-19. Children account for approximately 5% of the total patients diagnosed with COVID-19, and the disease in children is mild, with less than 1% of hospital admissions.^10^ As the disease in children is generally mild, this could be the plausible explanation to the fact that leukopenia is not found in significant pediatric patients.

In our study mean age of children was 7.0±4.3 years comparable with mean age of seven years in a study from China and a multi-organizational study of Global Health Network.^11^,^12^ Many other studies showed a significantly lower mean age of three years.^13^,^14^ In the studies in which the mean age of children was less than five years was due to the reason that children had milder disease and they were without any comorbid condition. There was a male preponderance 51 (61%) consistent with few studies.^12^,^15^ The cause of male predominance is unclear but partly it may be due to outdoor playing activities of male children as compared to girls leading to increased risk of exposure.

In COVID group mean leukocyte count was 14.0 ± 12.5 x10^9^/L and mean lymphocyte count was 39.1 ± 21.4%. This is consistent with a study in Wuhan, China where lymphopenia was not found in pediatric patients.^16^ Patients with MIS-C/ KD showed significant neutrophilia 76.5 ± 15.0%. These results are consistent with a study from Europe where significant increased neutrophil counts were found in MIS-C patients.^17^

Literature on COVID-19 in children is scarce as they generally have mild disease. In our study our two third children had mild to moderate disease. As compared to our results a study from China showed 90% children were either asymptomatic or had mild to moderate disease.^11^ In our study a significant proportion of children were diagnosed as post COVID MIS-C/KD which was reported in Lancet.^18^

In our study, median NLR was 1.96 as majority of our children were in mild-moderate category and in severe-critical group it was 5.26. This is in contrast to a study conducted in Italy where median NLR was 4.5 but same in the aspect that rising NLR was independent biomarker of disease severity and poor outcome.^19^ Our results were synchronized with a study conducted in Karachi where rising NLR was directly related with poor prognosis.^20^ In another study conducted in Jinnah hospital Lahore high NLR was associated with mortality in COVID infected patients.^21^

### Limitations & Strengths:

This was a single centered study limiting the generalization of our results. However, the Children’s Hospital Lahore is the largest tertiary care hospital for children in Pakistan reflecting a comprehensive picture of COVID-19 disease spectrum in children and being a referral center, we receive children with multiple comorbid conditions from all over Punjab. Secondly this is the first study for looking into the association of disease severity of COVID-19 in children with blood counts.

## CONCLUSION

Leukopenia and lymphopenia as per our study is not a feature of COVID-19 in children as most of our children presented in mild to moderate category but increased NLR was a prognostic factor which can be used independently to assess the severity and prognosis of clinical symptoms in COVID-19 patients. More children with severe COVID had lower lymphocyte count as compared to mild to moderate disease. The presence of an underlying comorbid condition is a significant risk factor for poor outcome.

### Authors’ Contribution:

**AB:** Conceived, designed, manuscript writing, accuracy & integrity of work.

**AC:** Data collection, contributed in manuscript writing.

**IB:** Proof reading.

**NS:** Laboratory Data & critical review.
